# Field cancerization therapy with ingenol mebutate contributes to restoring skin-metabolism to normal-state in patients with actinic keratosis: a metabolomic analysis

**DOI:** 10.1038/s41598-019-47984-x

**Published:** 2019-08-08

**Authors:** Valeria Righi, Elisabetta Tarentini, Adele Mucci, Camilla Reggiani, Maria Cecilia Rossi, Federica Ferrari, Alice Casari, Cristina Magnoni

**Affiliations:** 1 Dipartimento di Scienze per la Qualità della Vita, Università di Bologna, Campus Rimini, Corso D’Augusto 237, 47921 Rimini, Italy; 2 Dipartimento Chirurgico, Medico, Odontoiatrico e di Scienze Morfologiche con interesse Trapiantologico, Oncologico e di Medicina Rigenerativa, Università di Modena e Reggio Emilia, via del Pozzo 71, 41124 Modena, Italy; 3 Dipartimento di Scienze Chimiche e Geologiche, Università di Modena e Reggio Emilia, via G. Campi 103, 41125 Modena, Italy; 4 Centro Interdipartimentale Grandi Strumenti, Università di Modena e Reggio Emilia, via G. Campi 213/A, 41125 Modena, Italy

**Keywords:** Metabolomics, Medical research

## Abstract

Actinic keratosis (AK) is a skin premalignant lesion, which progresses into squamous cell carcinoma (SCC) if left untreated. Ingenol mebutate gel is approved for local treatment of non-hyperkeratotic, non-hypertrophic AK; it also has the potential to act as a field cancerization therapy to prevent the progression of AK to SCC. To gain better insights into the mechanisms of ingenol mebutate beyond the mere clinical assessment, we investigated, for the first time, the metabolome of skin tissues from patients with AK, before and after ingenol mebutate treatment, with high-resolution magic angle spinning nuclear magnetic resonance spectroscopy. The metabolomic profiles were compared with those of tissues from healthy volunteers. Overall, we identified a number of metabolites, the homeostasis of which became altered during the process of tumorigenesis from healthy skin to AK, and was restored, at least partially, by ingenol mebutate therapy. These metabolites may help to attain a better understanding of keratinocyte metabolism and to unmask the metabolic pathways related to cell proliferation. These results provide helpful information to identify biomarkers with prognostic and therapeutic significance in AK, and suggest that field cancerization therapy with ingenol mebutate may contribute to restore skin metabolism to a normal state in patients with AK.

## Introduction

Actinic keratosis (AK) is a cutaneous intraepithelial neoplastic lesion that typically develops on sun-damaged skin of elderly individuals. AK is the most frequent pre-malignant skin lesion that occurs in humans, and its incidence is increasing worldwide^1^. It is a pre-cancerous lesion of the skin that is histopathologically characterized by atypia and disorderly maturation of keratinocytes. The atypical keratinocytes can be localized in the basal cell layer of epidermis (AK grade I) or can be placed in the epidermal granular and/or spinous layer (AK grade II) or can involve all the layers of epidermis up to the corneum layer (AK grade III). The presence of hyperkeratosis and parakeratosis is a common histopathological finding in AK. It could be present an atrophy of the epidermal surface. Elastosis and a chronic inflammatory infiltrate is commonly present in the dermis. AK can progress into squamous cell carcinoma (SCC) if left untreated. The mechanism of this process is yet unknown, even when chronic ultraviolet (UV) exposure is considered to be the main risk factor^2,3^. The rate of progression ranges from 0.025% to 20%; the high variability of this is due to different definitions of AK applied in different studies^3^.

Distinct AKs differ in their genetic and biochemical characteristics^4^. UV radiation affects not only DNA, but also membrane phospholipids and the histidine derivative trans-urocanic acid (trans-UCA), which leads to the production of membrane lipid-derived mediators, such as arachidonic acid and platelet-activating factor (PAF), and the photo-isomerization of UCA into *cis*-UCA, which together alter intracellular signaling. Moreover, the UV-mediated dysregulation of the cytokine milieu leads to inflammation, changes in T-cell homeostasis, and immunosuppression that may contribute to keratinocyte transformation^5,6^. Moreover, UVB induces a mutation of *TP53* gene, resulting in irreversible inactivation of the tumor suppressor feature of the protein, which potentially leads to the progression to SCC. UV radiation has been observed to induce apoptosis in keratinocytes with one mutation in *TP53*. However, additional *TP53* inactivation renders the keratinocytes resistant to apoptosis and consequently uncontrolled proliferation, which is one of the early events in the progression of AK to SCC^3,7^. Of note, the progression of individual AKs cannot be predicted^4^. Therefore, all AK lesions, regardless of their grade, should be carefully monitored and treated. Indeed, proper treatment of AK lesions can reduce the risk of progression^1^.

Ingenol mebutate gel is an approved treatment option for cancerization field and non-hyperkeratotic, non-hypertrophic AK^8^. Several studies have shown that this topical agent is effective and cost-saving, with an acceptable tolerability profile^9–15^. Ingenol mebutate has the potential to prevent the progression of AK to SCC^16^; however, its efficacy has mainly been assessed based on clinical outcomes^17^, while histological studies on its effects of ingenol mebutate on skin metabolites (also over a long-term period) are lacking.

Metabolomics is the global and systematic assessment of metabolites existing in a biological system, aimed at selecting potential predictive disease biomarkers that are able to provide insights into the underlying pathophysiology. Metabolomic approaches have been already successfully used in the dermatology field^18^. Nuclear magnetic resonance (NMR) spectroscopy represents an important tool for the analysis of the fingerprint of tissue. *Ex vivo* high-resolution magic angle spinning (HR-MAS) NMR can detect the biochemical tissue composition, which identifies potential biomarkers to differentiate healthy tissue from pathological tissue, and can detect correlations between certain metabolites and proliferative markers. However, to our knowledge, this technique and the related approach has never been exploited to study AK specimens.

The purpose of this study was performing a comprehensive skin histopathological and metabolomics analysis to identify potential biomarkers of AK, and to investigate the effects of field cancerization treatment with ingenol mebutate on metaboloma.

## Results

### Clinical and histopathological analysis

**Figure 1:**
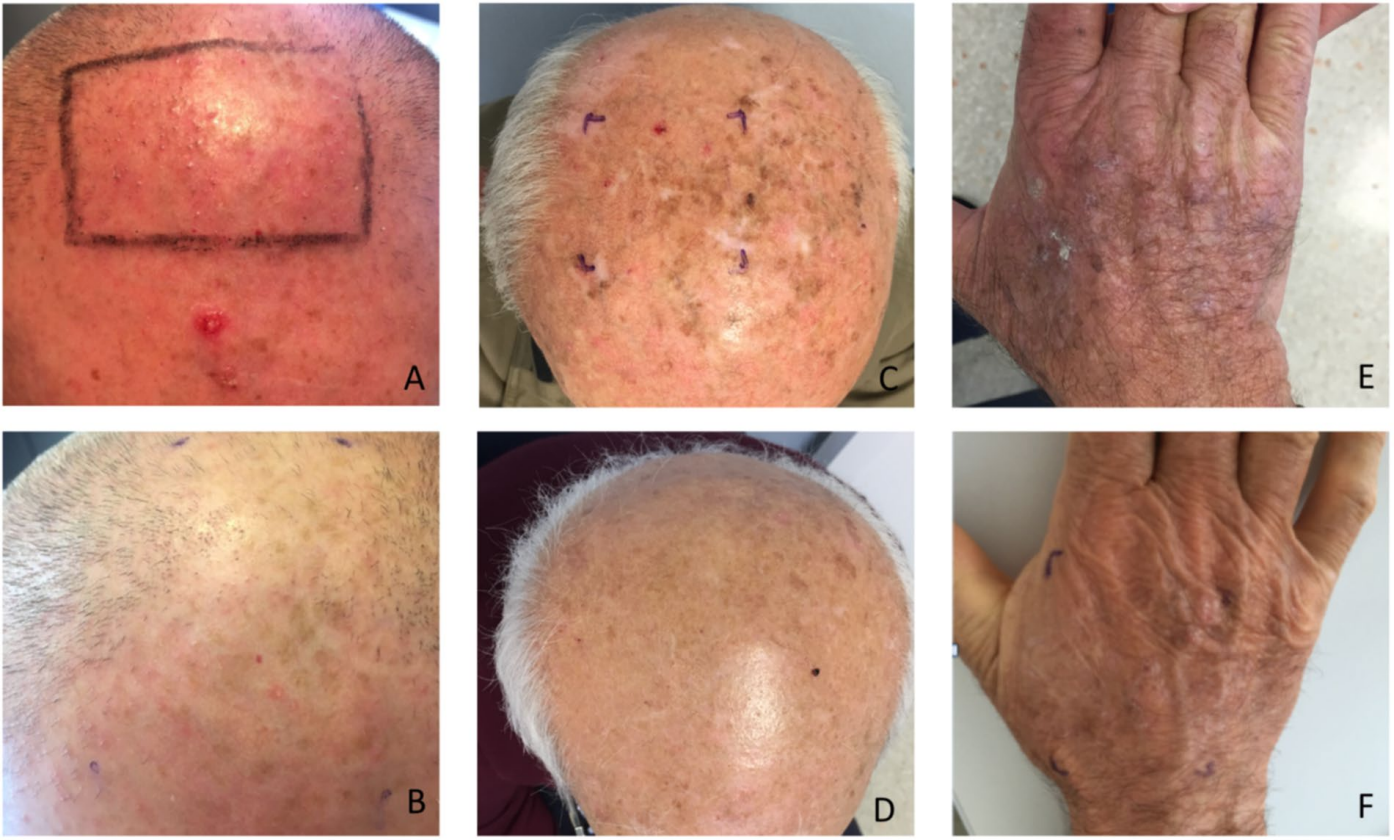
Clinical images of AK before and after treatment with ingenol mebutate. (**A**) A patient affected by multiple AK grade I of the scalp pre-treatment. (**B**) The same patient after treatment. AK grade II of the scalp (**C**) pre-treatment and (**D**) after treatment. AK grade III of the hand (**E**) pre-treatment and (**F**) after treatment.

**Figure 2:**
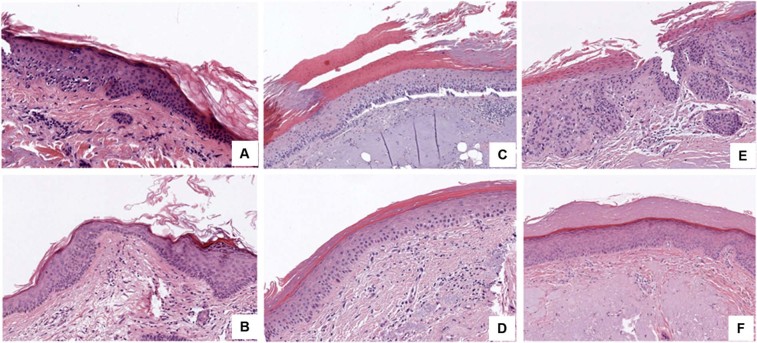
(**A**) A histological image of AK grade I before the treatment with ingenol mebutate, which demonstrates mild atypic keratinocytes in the lower layers of epidermis. (**B**) A histological image of complete clearance of AK grade I after the treatment with ingenol mebutate. (**C**) A histological image of AK grade II before the treatment with ingenol mebutate, which demonstrates an increased number of keratinocytes and morphological atypic cells in the basal and spinous layer of epidermis. (**D**) A histological image of complete clearance of AK grade II after the treatment with ingenol mebutate. (**E**) A histological image of AK grade III before the treatment with ingenol mebutate, which shows severe atypic cells in all the layers of the epidermis, parakeratosis, and elastosis. (**F**) A histological image of complete clearance of AK grade III after the treatment with ingenol mebutate, which shows a sharp improvement of the atypia of the the keratinocytes, clearance of the parakeratosis. Elastosis is still present after the treatment.

A total of 21 subjects were evaluated, which included 11 patients with AK and ten healthy individuals. Most of the lesions (7/11; 63%) showed an improvement of the grade of AK upon treatment with ingenol mebutate. Complete clearance was observed after treatment in five cases (45%). Histopathologic features remained stable in four cases (36%). Figures 1 and 2 show some relevant clinical and histopathological images.

In all cases, where an improvement of the AK grade was observed, a concomitant morphologic improvement was also reported. In the five cases with complete clearance after ingenol mebutate treatment, the total number of cells and mitoses was increased. On the other hand, in the four cases in which stable histopathologic features were determined, the level of atypia observed at the morphologic analysis was consistently maintained.

Parakeratosis, which was demonstrated in ten out of 11 cases before treatment, improved in eight cases (80% of those with parakeratosis) after ingenol mebutate therapy. In two cases, the histopathology analysis detected acantolysis, which resulted in improvement after the treatment in both cases. Nine lesions were hyperplastic. Loss of this feature after treatment was observed in six cases (54%).

### Characterization of the metabolites

**Figure 3:**
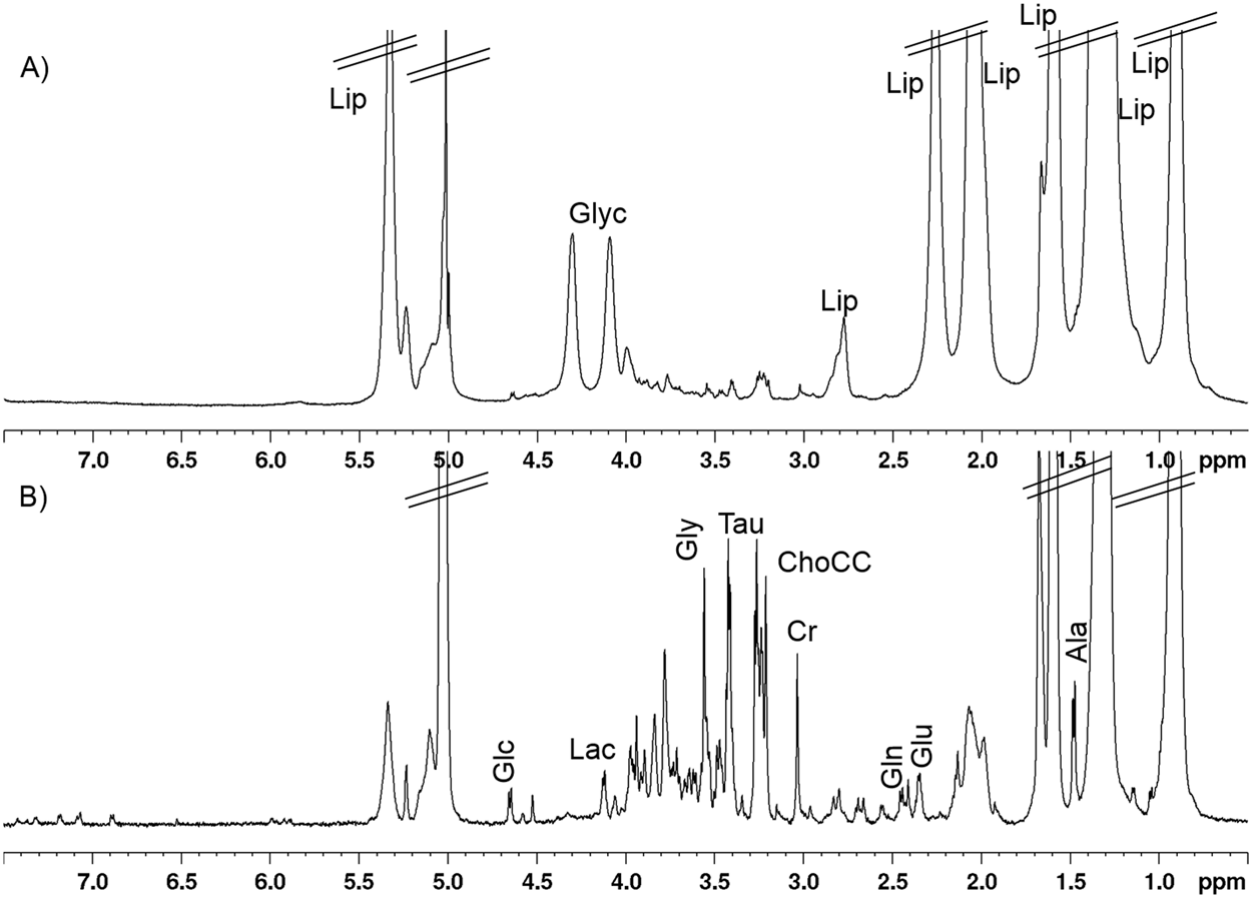
Representative HR-MAS 1D ^1^H NMR spectra of a healthy skin tissue sample. (**A**) 1D ^1^H water pre-saturated spectrum and (**B**) 1D ^1^H CPMG spectrum. Major metabolites are labelled: Ala, alanine; ChoCC, choline containing compound; Cr, creatine; Glc, glucose; Gln, glutamine; Glu, glutamate; Gly, glycine; Glyc, glycerol; Lac, lactate; Lip, lipids and Tau, taurine.

Representative *ex vivo* HR-MAS 1D ^1^H NMR spectra of a healthy skin tissue are shown in Fig. 3. Figure 3A shows the conventional 1D ^1^H water pre-saturated spectrum and Fig. 3B shows the 1D ^1^H Carr–Purcell–Meiboom–Gill (CPMG) spectrum from a healthy tissue sample. In the 1D ^1^H water pre-saturated spectrum (Fig. 3A), highlighting both narrow and broad signals, the predominant resonances were due to lipids (Lip) and bound glycerol (Glyc), indicating that the main metabolites were triglycerides. The 1D ^1^H CPMG spectrum (Fig. 3B), where broad signals are partially reduced, displays the presence of amino acids (alanine [Ala], glutamine [Gln], glutamate [Glu], pyroglutamic acid [PGA], and glycine [Gly]), osmolites (lactate [Lac], taurine [Tau], creatine [Cr], choline-containing compounds [ChoCC]), and glucose (Glc).

The assignment of metabolites is based on the analysis of 1D ^1^H, and selected 2D (COSY, TOCSY, and HSQC) NMR spectra (see the ‘Methods’ section). The obtained data are compared with those from the literature and, where possible, with the experimental spectra reported in the Human Metabolome Database (http://www.hmdb.ca/). The list of identified metabolites is reported in Table S1.

### Metabolic profiles of normal, AK, and treated AK samples

**Figure 4:**
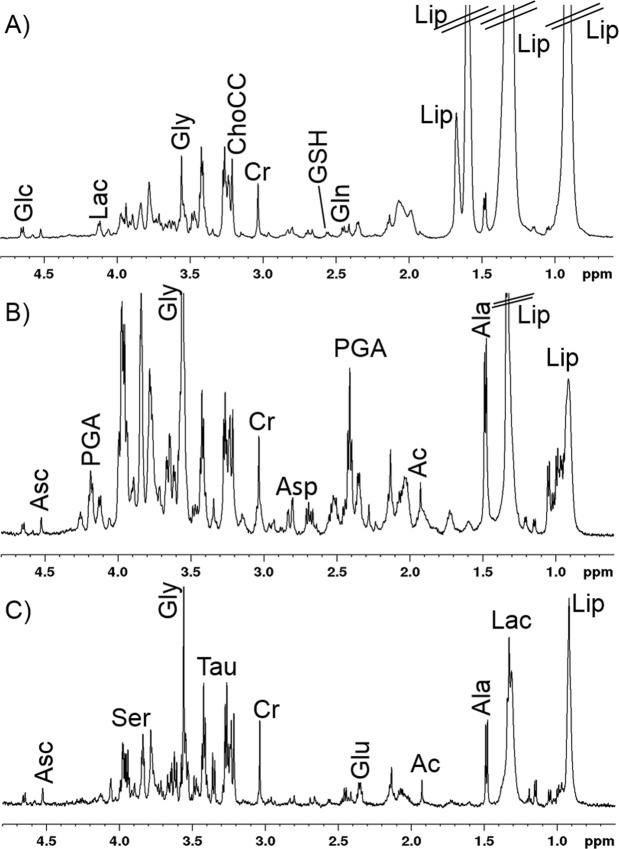
1D ^1^H CPMG spectra from: (**A**) healthy, (**B**) actinic keratosis, and (**C**) treated actinic keratosis tissue samples (**B** and **C** tissue samples are from the same patient). Visible metabolites are labelled: Ac, acetate, Ala, alanine; Asc, ascorbate; ChoCC, choline containing compound; Cr, creatine; Glc, glucose; Gln, glutamine; Glu, glutamate; Gly, glycine; Glyc, glycerol; GSH, glutathione; Lac, lactate; Lip, lipids; PGA, pyroglutamic acid; Ser, serine and Tau, taurine.

To gather insights into the metabolism of the skin in samples from healthy subjects, AK patients, and patients with AK treated with ingenol mebutate (tAK), the related ^1^H CPMG NMR spectra were compared (Fig. 4). A long total echo-times (360 ms) was used to reduce the lipid resonances and to enhance the signals from metabolites. Nevertheless, some lipid residual signals remained in the CMPG spectra (Fig. 4A).

The inspection of the spectra allowed us to observe some trends in the metabolic profile of the three different tissues. In particular, the amount of lipids was generally higher in the samples from healthy subjects with respect to the AK and tAK samples, whereas the levels of Gly, Tau, and PGA seemed higher in AK compared with samples from healthy subjects and tAK subjects.

### Chemometric analysis

**Figure 5:**
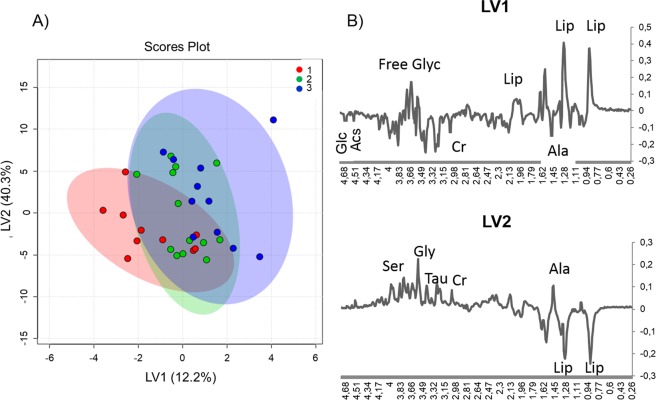
Partial least square discriminant analysis (PLS-DA) of the spectral profiles. (**A**) Latent variables 1 (LV1) and 2 (LV2) scores plot showing (1) healthy skin, (2) AK, and (3) tAK with ingenol mebutate samples. (**B**) PL-SDA loadings profiles of LV1 and LV2.

First, an unsupervised multivariate analysis on the complete spectral profiles of the three types of tissues was performed. Principal component analysis (PCA) did not show any substantial clustering. Next, we ran a partial least square discriminant analysis (PLS-DA). In this case, the scores plot shows that the three groups cluster only partially and that the scores move to negative values of the latent variables 1 (LV1) and 2 (LV2), passing from healthy, to AK, and to tAK samples (Fig. 5A and Fig. S1).

Inspection of the loadings profiles (Fig. 5B) points to some metabolites as the main responsible of the metabolic changes: ascorbate (Asc), Glc, Ser, Cr, Gly, Tau, Lip, and free glycerol, showing that several metabolic changes accompany the transition from normal tissues towards AK, and tAK samples. To evaluate the alterations during this transformation and then the skin recovery after treatment (tAK samples), we moved towards a more quantitative analysis, focusing on the metabolites, the signals of which can be reliably deconvoluted.

### Metabolites quantification

**Figure 6:**
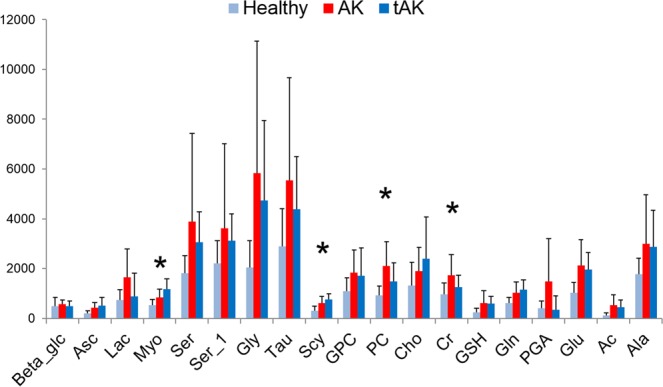
Bar plots showing the relative amounts (mean ± standard errors) of the selected metabolites from deconvoluted NMR signals. Statistical significant metabolites (p < 0.05), Myo, Scy, Cr, and PC detected by one-way ANOVA are labelled with asterisks, for details see also table in Fig. S2.

Figure 6 reports the relative amounts of selected metabolites obtained from deconvoluted NMR signals. According to this analysis, we observed that the amount of most of the metabolites, such as Lac, Ser, Gly, Tau, glyceryl phosphorylcholine (GPC), phosphorylcholine (PC), Cr, glutathione (GSH), PGA, Glu, acetate (Ac), and Ala, apparently increased during the development of AK, but decreased upon treatment with ingenol mebutate. Contrary to this, a completely different trend was observed for other metabolites, such as Asc, Myo, Scy, Cho, and Gln. Their amounts increased from healthy to AK, and further augmented in tAK. ANOVA (Fig. S2) demonstrated that the differences of the values obtained for Myo were statistically significant when the tAK class was compared with AK and healthy classes. The differences of Scy values were statistically significant when the healthy class was compared with AK and tAK classes, whereas the variations of the Cr values were significant when the AK class was compared with the healthy and tAK ones, and finally, PC differences were significant when the healthy class was compared with the AK class.

**Figure 7:**
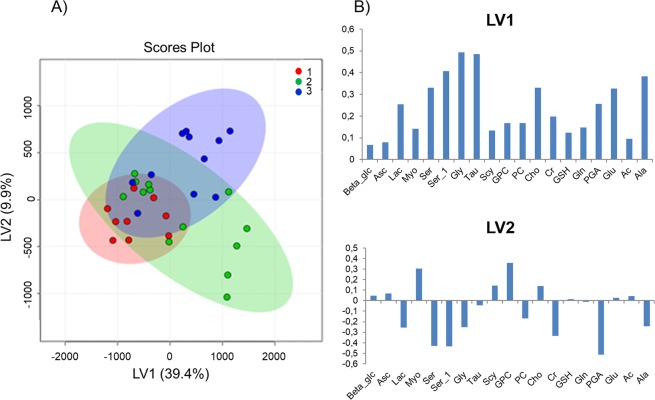
Partial least square discriminant analysis (PLS-DA) of the deconvoluted signals. (**A**) Latent variables 1 (LV1) and 2 (LV2) scores plot showing (1) healthy, (2) AK, and (3) tAK samples. (**B**) PL-SDA loadings profiles of LV1 and LV2.

The PCA analysis on the deconvoluted signals showed no clear clustering for the three groups; therefore, we performed the PLS-DA analysis (Fig. 7). The PLS-DA scores plot (Fig. 7A) demonstrates a discrimination among the three classes (Fig. S3), which was better than that obtained by analyzing the spectral profiles. The scores of AK and tAK samples were characterized by positive values of LV1, whereas the scores of the healthy class were distinguished by negative values of LV1. With regards to the LV2, healthy and AK samples scores were characterized by negative values, whereas tAK scores were clearly distinguished as positive values (Fig. 7A). The loadings plots (Fig. 7B) demonstrate that LV1 mainly reflects the enhancement of metabolite concentrations from healthy to AK, and to tAK samples. The loadings of LV2 point, instead, to two groups of important metabolites: Ser, Gly, Ala, Lac, PC, Cr, and PGA (negative values), and Asc, Myo, GPC, GSH, Scy, and Cho (positive values).

Based on these analyses, two major groups of metabolites can be identified: the group including Ser, Gly, Tau, Glu, and Lac enhancing from healthy to AK class and subsequently decreasing in the tAK class, and the group including Asc, Myo, Scy, Cho, and Gln increasing their amounts from healthy to AK, and to tAK classes. Noteworthy, due to the low number of samples included in these analyses, some of these variations are to be considered as probable trends.

## Discussion

The use of metabolomics in the clinical setting aims to identify the metabolic profile of healthy subjects and patients, also in response to therapeutic interventions. This application of precision medicine enables both the identification of prognostic biomarkers for specific diseases, and the monitoring of treatment efficacy in several conditions, including cancer. In addition, the systematic analysis of individual small-molecule profiles allows metabolomics to provide essential information for the establishment of therapies tailored to single patients^19,20^. AK is the most frequent pre-malignant skin lesion, which may progress into SCC, and ingenol mebutate has been demonstrated to be effective in improving the clinical outcome of AK, with total clearance in a high percentage of patients^14^.

However, metabolomic studies on the effects of the treatment with ingenol mebutate in AK are currently lacking. Therefore, we conducted a comprehensive skin histopathological and metabolomics analysis to identify potential biomarkers of AK, and investigated the potential metabolomic effects of field cancerization treatment with ingenol mebutate.

First, although we examined a limited number of patients in this study, we observed a major improvement in the samples collected 60 days after the treatment with ingenol mebutate at the histological level. No cases of worsening occurred, and complete resolution of the lesion was observed in almost 40% of the cases. These findings paralleled the reduction in the number of mitoses and by the general improvement in morphology. These findings support the effectiveness of ingenol mebutate in clinical practice for the treatment of AK.

With respect to the metabolomic analysis, the assessment of the metabolomic profile of skin samples from healthy subjects and from patients with AK showed that the amount of all the evaluated metabolites increased in the AK group. This suggests a profound modification of skin metabolism during the course of AK. However, following field cancerization treatment with ingenol mebutate, some of the metabolites decreased in quantity, while others maintained higher levels compared with the healthy group. Therefore, two distinct classes of metabolites were identified in tAK samples.

Ser, Gly, PGA, Tau, Glu, and Lac decreased in concentration after ingenol mebutate treatment. Among these metabolites, PGA is worth particular attention because this molecule is involved in a number of metabolic pathways, including the calcium-dependent transglutaminases critical to the condition of the skin, (*e.g*. through filaggrin metabolism)^21^ and pyroglutamic acid synthetase involved in folate homeostasis and antifolate resistance in cancer^22^. On the other hand, the increase of Gly during the progression of AK may be associated with rapid cell proliferation, as this metabolite is a crucial component of purine metabolism in rapidly proliferating cells^23^. Gly is also largely present in collagen that supports major remodeling processes in the skin during SCC tumorigenesis^24^. A metabolomics signature of sun-exposed skin tissues revealed that high oxidative stress is responsible for the imbalance of GSH metabolism, impairing its role as a scavenger of free oxygen species and that Glu and Ser are metabolites involved in the Gly and GSH pathways^25^. Tau plays a role in the maintenance of the intracellular osmotic equilibrium^26^. Epidermal keratinocytes pursue an osmolyte strategy, accumulating the osmolyte Tau when exposed to hyperosmotic stress^27^. Tau prevents the abnormal production and release of the immunosuppressive platelet-activating factor (PAF) from membranes of epidermal cells exposed to UVB radiation, protecting against UVB-induced immunosuppression^28^.

Even if Lac is a secondary metabolite, its presence in the class of metabolites affected by the ingenol mebutate therapy is of particular interest, because of the critical role played by this metabolite in the long-known Warburg effect, a switch from oxidative phosphorylation to aerobic glycolysis, which is a hall mark of cancer progression and maintenance of the transformed phenotype^29,30^.

Reprogramming of metabolism has an emerging role in the understanding and – potentially – treatment of cancer^31,32^.

The amount of reduction of these metabolites after ingenol mebutate therapy suggests that this drug may induce AK cells to recover the metabolome typical of the healthy skin, favoring a *restitutio ad integrum* (normalization) of the metabolic pathways altered by the tumorigenic process.

The class of metabolites with concentrations maintained high or even increased after ingenol mebutate therapy are: Asc, Myo, Scy, Cho, and Gln. This finding seems somehow counterintuitive and requires further investigations; however, some preliminary explanations can be found by going deeper into the nature and the biological effects of these metabolites.

Asc, or vitamin C, exerts a powerful antioxidant protection in several tissues and circumstances, including in UV-exposed skin, with anti-inflammatory and anti-apoptotic effects. Moreover, Asc may also influence morphology and differentiation of keratinocytes, as well as gene expression in the skin. The structure of the stratum corneum is potentiated, with an increase of keratohyalin granules in number and of barrier lipids. The expression of differentiation markers pro-filaggrin and filaggrin and the stabilization of hypoxia-inducible factor-1 (HIF-1) reflect the positive effect of vitamin C on tissue remodeling and keratinocytes cell survival at molecular level^33^. Therefore, the increased concentration of Asc reported during the study may indicate remodeling of the skin. On the other hand, Myo is the precursor of several phosphate derivatives (mainly phosphatidylinositols), which exert several important functions in cellular metabolism and signaling. By mediating the regulation of the permeability of ion channels and the signal transduction of some membrane receptors (including the insulin receptor) these myo-derivatives can induce gene transcription, mRNA export and protein synthesis involved in many intracellular functions, such as embryonic development, cell proliferation, and stress response^34^. Interestingly, the naturally occurring plant antioxidant inositol hexaphosphate (IP6) and its precursor, inositol, have been shown to enhance the efficacy of chemotherapy by exerting immunostimulatory and antioxidant effects, which may even control metastasis^35^. ChoCC are nutrients with an essential precursor role in the synthesis of membrane phospholipids^36^ and the elevated ChoCC concentration also after therapy with ingenol mebutate strongly suggests that these metabolites were involved in enhanced tissue remodeling.

Finally, as in the case of Gly, a major component of collagen, a high level of Gln was found in AK samples compared with the healthy controls. However, Gln concentration further increased upon ingenol mebutate treatment. In view of the role of Gln in promoting collagen biosynthesis^37^, the elevated concentration of this amino acid suggests that intense tissue remodeling may be required to counteract the AK transformed phenotype at a histological level, and that this may be one of the mechanisms of action of ingenol mebutate. Since we have analyzed the samples before treatment and 60 days after treatment, we believe that, the results observed after treatment are derived from metabolic changes induced by ingenol mebutate. However, it is worth noticing that ingenol mebutate induces a tumor cell-directed inflammatory response^38,39^, and therefore the improvement in the metabolic profile may be due, at least in part, to the activation of the inflammatory responses and cytokines.

In conclusion, our first metabolomic survey in skin tissues affected by AK allowed the identification of small-molecule metabolites, the homeostasis of which became altered during the process of tumorigenesis from healthy skin to AK. These results may help to gain a better understanding of keratinocyte metabolism and to unmask the metabolic pathways related to cell proliferation. Even if the relatively small number of cases examined represents a limitation of this study, the metabolome analysis in samples from the same patients with AK after a 2-month therapy with ingenol mebutate provides information relevant to the discovery of biomarkers of drug responsiveness, as well as further insight on the mechanism of action of this promising therapeutic agent for AK, which also represents an invaluable tool for the prevention of progression to SCC. Our results pave the way for future similar studies, in larger samples, aimed at further investigating the effects of different treatment on the metabolomic profile of skin lesions.

## Methods

### Study setting and design

This study was conducted at the Policlinico of Modena (Modena, Italy), from February 2016 to August 2017. The project consisted of two main phases, with the following objectives: (i) define the metabolic profile of tissues from patients with AK, compared with that of normal tissues from healthy volunteers, with the potential identification of potential AK biomarkers by NMR analysis; (ii) monitor the metabolic evolution of AK after treatment with ingenol mebutate and photodynamic therapy. Treatment with ingenol mebutate consisted of topical application for 3 days (1 vial/day), in an inpatient setting.

The study was conducted according to the Helsinki declaration, after approval of the study design by the local Ethical Committee (Comitato Etico dell’Area Vasta Emilia Nord, Policlinico di Modena, Via Largo del Pozzo 71, 41124). All patients signed an informed consent form before the inclusion in the study.

### Study sample

In total, 23 subjects were enrolled: ten healthy subjects undergoing preemptive routine skin surgery for pigmented benign lesion removed for aesthetic reasons (e.g. dermal nevi), and 13 consecutive patients with AK who were eligible to therapy with ingenol mebutate (11) and photodynamic therapy (2) in a routine clinical practice setting. The samples from AK patients were taken from the same area before and after treatment. Samples from healthy subjects were taken from a sun-unexposed area in order to limit any bias due to photodamage. Samples treated with photodynamic therapy were removed from the analysis in order to not introduce a bias.

Samples from untreated patients with AK (AK group) were collected from an area of field cancerization that showed more than five AK lesions. Field cancerization was determined following the recommendation of the European Dermatology Forum^40^ and was defined as one body region showing at least six AK lesions and contiguous areas of chronic actinic sun damage and hyperkeratosis. Samples from the same patients with AK were also collected 60 days after treatment (tAK group). All samples were collected by punch biopsy of 6 mm diameter, dissected to separate skin from adipose tissue and directly frozen in liquid nitrogen. The samples were stored at −80 °C until analysis.

### Histopathology assessment

The histopathological features examined in each case were the grade of AK (presence or absence of AK lesions and – in case of lesion evidence – the level of involvement of the epidermis was evaluated with the following scale: 1 – only basal layer involved, 2 – two/three of the epidermis involved from the lowest level, 3 – full thickness involvement), and the presence or absence of the following conditions: parakeratosis, orthokeratosis, acantholysis, clear cells, hyperplasia, elastosis, atrophy, hypertrophy, ulceration, and pigmentation. In addition, the extent of the inflammatory infiltrate (low, medium and high), the presence of contingent close lesions (e.g. solar lentigo, seborrheic keratosis), the level of atypia, evaluated as morphological atypia (1 – low, 2 – medium and 3 – high), the increased number of cells (1 – low, 2 – medium and 3 – high) and the number of mitoses (1 – low, 2 – medium and 3 – high) were also evaluated.

### HR-MAS NMR

Tissue was introduced in a 50 µl MAS zirconia rotor (4 mm OD) with 10 µl of deuterated water (D_2_O), closed with a cylindrical insert to increase sample homogeneity, then transferred into the probe cooled to 5 °C. HR-MAS NMR spectra were recorded with a Bruker Avance III HD 600 MHz spectrometer, operating at 600.13 and 150.90 MHz, for ^1^H and ^13^C, respectively. The instrument was equipped with a ^1^H, ^13^C HR-MAS probe, which temperature was controlled by a Bruker Cooling Unit. All experiments were performed at 5 °C to prevent tissue degradation processes^41^. Samples were spun at 4000 Hz. After the set up (about 20 min), two different types of one-dimensional (1D) proton spectra were acquired by using: (i) a composite pulse sequence (zgcppr), with 4 s water-pre-saturation during relaxation delay, 8 kHz spectral width, 32 k data points, 32 scans; (ii) a water-suppressed spin-echo CPMG sequence (cpmgpr) with 4 s water pre-saturation during relaxation delay, 1 ms echo time (τ), and 360 ms total spin–spin relaxation delay (2 nτ), 8 kHz spectral width, 32 k data points, 128 scans. 2D COSY spectra were acquired using a standard pulse sequence (cosygpprqf) and 0.5 s water pre-saturation during relaxation delay, 8 kHz spectral width, 4 k data points, 32 scans per increment, 256 increments. 2D ^1^H, ^1^H-TOtal Correlation SpectroscopY (TOCSY) spectra were acquired using a standard pulse sequence (mlevphpr) and 0.5 s water-pre-saturation during relaxation delay, 100 ms mixing (spin-lock) time, 4 kHz spectral width, 4 k data points, 32 scans per increment, 128 increments. 2D ^1^H, ^13^C-heteronuclear single quantum coherence (HSQC) spectra were acquired using an echo–antiecho phase-sensitive standard pulse sequence (hsqcetgp) and 0.5 s relaxation delay, 1.725 ms evolution time, 4 kHz spectral width in f2, 4k data points, 128 scans per increment, 17 kHz spectral width in f1, 256 increments^41^.

### Data processing and bioinformatics

Clinical and histopathological data were analyzed by descriptive statistics. The HR-MAS spectra were analyzed with two different methods to determine whether there were differences between the three experimental groups. First, chemometric methods were used to compare the differences in the spectral profiles from each group. In the second approach, the relative concentrations of 18 specific metabolites were estimated in CPMG using the areas of selected peaks spectra. In both analyses, we used Mnova 11 software (MestReNova, ver. 11. 0, 4-18998, 2017 Mestrelab Research S. L., Santiago de Compostela, Spain)^42^.

In the first case, the ^1^H CMPG NMR spectra obtained for each tissues sample were baseline correct, normalized with respect to sample weight and binned (δ 0.01 ppm), then the whole 4.8–0.5 ppm region was analyzed through PCA and PLS-DA.

In the second case, the areas of selected peaks of CPMG spectra were obtained with an automated/controlled fitting routine based on the Levenberg-Marquardt algorithm applied after manual peak selection, adjusting peak positions, intensities, line widths and Lorentzian/Gaussian ratios, until the residual spectrum was minimized^43^. Data were reported as means ± standard errors (arbitrary units). For Student’s t-test, paired two-sample test was used to determine the means. A p-value < 0.05 was considered statistically significant.

ANOVA, PCA and PLSDA analyses were performed using MetaboAnalyst 4.0 – a comprehensive server for metabolomic data analysis^42^. Pareto scaling was used to make individual features more comparable to adjust for the differences among the samples.

## Supplementary information

Supporting Information
