# Supplementary material for: Field cancerization therapy with ingenol mebutate contributes to restoring skin-metabolism to normal-state in patients with actinic keratosis: a metabolomic analysis
Source: Sci Rep. 2019 Aug 8;9:11515. doi: 10.1038/s41598-019-47984-x (PMC6687779; doi:10.1038/s41598-019-47984-x)

**Supporting Information**

**Field cancerization therapy with ingenol mebutate contributes to restoring skin-metabolism to normal-state in patients with actinic keratosis: a metabolomic analysis**

Valeria Righi^1,*,+^, Elisabetta Tarentini^2^, Adele Mucci^3^, Camilla Reggiani^2^, Maria Cecilia Rossi^4^, Federica Ferrari^2^, Alice Casari^2^, Cristina Magnoni^2,+^

^1^Dipartimento di Scienze per la Qualità della Vita, Università di Bologna, Campus Rimini, Corso D’Augusto 237, 47921 Rimini, Italy; ^2^Dipartimento Chirurgico, Medico, Odontoiatrico e di Scienze Morfologiche con interesse Trapiantologico, Oncologico e di Medicina Rigenerativa, Università di Modena e Reggio Emilia, via del Pozzo 71, 41124 Modena, Italy; ^3^Dipartimento di Scienze Chimiche e Geologiche, Università di Modena e Reggio Emilia, via G. Campi 103, 41125 Modena, Italy; ^4^Centro Interdipartimentale Grandi Strumenti, Università di Modena e Reggio Emilia, via G. Campi 213/A, 41125 Modena Italy

^+^V.R. and C.M. contributed equally to this work.

**^*^ corrisponding authors:**

**Valeria Righi**

e-mail: [valeria.righi2@unibo.it](mailto:valeria.righi2@unibo.it)

Valeria Righi, PhD

Dipartimento di Scienze per la Qualità della Vita,

Università di Bologna, Campus Rimini,

Corso D’Augusto 237, 49921 Rimini, Italy

Table S1. List of ^1^H and ^13^C chemical Shift (δ, ppm) of metabolites. ^a^,^1^H chemical shifts refer to Ala doublet at 1.48 ppm; ^b^,^13^C chemical shifts refer to Ala at 16.8 ppm.

|  | **Metabolites** | **δ^1^H**^a^ | **δ^13^C**^b^ | **Assignment** |
| --- | --- | --- | --- | --- |
| **1** | Fatty acids | 0.89 | 16.5 | CH_3_ |
|  |  | 1.32–1.29 | 32.6–34.2 | (CH_2_)_n_ |
|  |  | 1.59 | 27.4 | CH_2_-CH_2_-C=O |
|  |  | 2.04 | 29.6 | CH_2_CH=CH |
|  |  | 2.27 | 36.1 | CH_2_-C=O |
|  |  | 2.78 | 27.9 | =CH-CH_2_-CH= |
|  |  | 5.32 | 128–130 | -CH=CH- |
| **2** | Lactate | 1.33 | 22.7 | CH_3_ |
|  |  | 4.12 | 71.3 | CH |
| **3** | Alanine | 1.48 | 19.3 | CH_3_ |
|  |  | 3.78 | c | α CH |
| **4** | Valine | 0.98 | 21.7 | γ CH_3_ (d) |
|  |  | 1.04 | 20.7 | γ CH_3_ (d) |
|  |  | 2.27 | 30.5 | β CH |
|  |  | 3.61 | c | α CH |
| **5** | Leucine | 0.96 | 24.9 | δ CH_3_ |
|  |  | 0.97 | 25.3 | δ CH_3_ |
|  |  | 1.70 | 42.4 | β CH_2_ |
|  |  | 1.73 | 28.8 | γ CH_2_ |
|  |  | 3.72 | c | α CH |
| **6** | Lysine | 1.88 | 32.9 | β CH_2_ |
|  |  | 1.47 | 22.6 | γ CH_2_ |
|  |  | 1.73 | 27.3 | δ CH_2_ |
|  |  | 3.03 | 39,7 | ε CH_2_ |
|  |  | 3.73 | c | α CH |
| **7** | Isoleucine | 1.97 | 25.2 | CH_3_ |
|  |  | 0.95–0.93 | 21 |  |
| **8** | Acetate | 1.92 | 26.6 | CH_3_ (s) |
| **9** | Glutamine | 2.11 | 27.3 | β CH_2_ |
|  |  | 2.44 | 31.4 | γ CH_2_ (td) |
|  |  | 3.71 | c | α CH |
| **10** | Glutamate | 2.36 | 34.3 | γ CH_2_ |
|  |  | 2.07 | 25.2 | β CH_2_ |
|  |  | 3.73 | c | α CH |
| **11** | Threonine | 1.32 | 21.8 | γ CH_3_ |
|  |  | 3.56 | 69 | β CH |
|  |  | 4.25 | 66.9 | α CH |
| **12** | α-glucose | 5.24 | 92.6 | 1 CH (d) |
|  |  | 3.55 | 72 | 2 CH |
|  |  | 3.72 | 73.1 | 3 CH |
|  |  | 3.42 | 76.4 | 4 CH |
|  |  | 3.82 | 71.9 | 6 CH_2_ |
| **13** | β-glucose | 4.64 | 96.4 | 1 CH (d) |
|  |  | 3.25 | 74.7 | 2 CH |
|  |  | 3.49 | 76.3 | 3 CH |
|  |  | 3.4 | 70.2 | 4 CH |
|  |  | 3.47 | 76.4 | 5 CH |
|  |  | 3.91 | 61.3 | 6 CH_2_ |
| **14** | Creatine | 3.06 | 39.6 | CH_3_ (s) |
|  |  | 3.95 | 56.4 | CH_2_ (s) |
| **15** | Histidine | 7.03 |  | 2 CH |
|  |  | 7.73 |  | 4 CH |
| **17** | UDP | 5.89–5.93 |  | CH |
|  |  | 7.91 |  | CH |
| **18** | Glycine | 3.56 | 44.3 | CH_2_ (s) |
| **19** | Taurine | 3.41 | 38.06 | N-CH_2_ (t) |
|  |  | 3.26 | 48.4 | S-CH_2_ |
| **20** | Tyrosine | 6.88 |  | 5 CH |
|  |  | 7.18 |  | 6 CH |
| **21** | Succinate | 2.37 | 34.2 | CH_2_ (s) |
| **22** | Glycerol bound | 4.08 | 62.2 | CH_2_ |
|  |  | 4.28 | 62.2 | CH_2_ |
|  |  | 5.22 | 69.4 | CH |
| **23** | Glyceryl phosphorylcholine | 4.32 | 60 | α’ CH_2_ |
|  |  | 3.67 | 66.6 | γ CH_2_ |
|  |  | 3.24 | 56.5 | N-CH_3_ (s) |
| **24** | Aspartate | 2.68–2.65 |  | CH_3_ |
|  |  | 3.90 |  | CH_2_ |
| **25** | Pyroglutamic acid | 2.03–2.51  2.41 | 25  30 | 2,4 CH_2_  3 CH_2_ |
|  |  | 4.19 | 59 | 1 CH |
| **26** | Myo-inositol | 3.53 | 72 | 1,3 CH |
|  |  | 4.06 | 71.3 | 2 CH |
|  |  | 3.63 | 73.1 | 4,6 CH |
|  |  | 3.27 | 74.4 | 5 CH |
| **27** | Scyllo-inositol | 3.36 | 73.6 | CH (s) |
| **28** | Serine | 3.87  3.94 | 61  57 | α CH  β CH_2_ |
| **29** | ETOH | 1.18  3.65 |  | CH_3_  CH_2_ |
| **30** | Glutathione | 2.55  2.95  4.57 |  | 5 CH_2_  4 CH_2_  1 CH |
| **31** | Ascorbate | 4.52 |  | 4 CH |
| **32** | Adenine | 8.23  8.35 |  |  |

Figure S1. PLS-DA cross validation details and permutation.


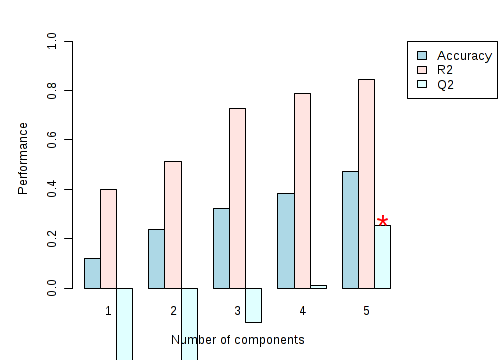


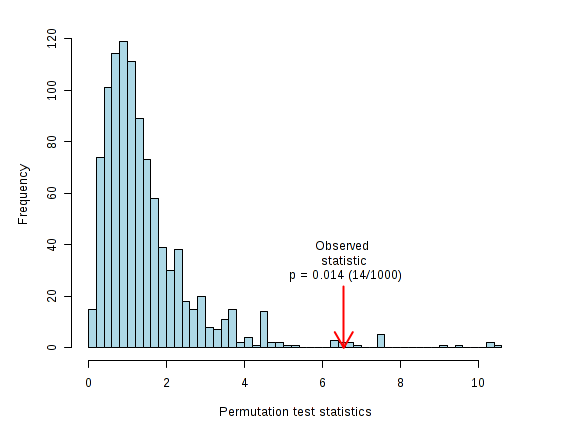


Figure S2. ANOVA results.


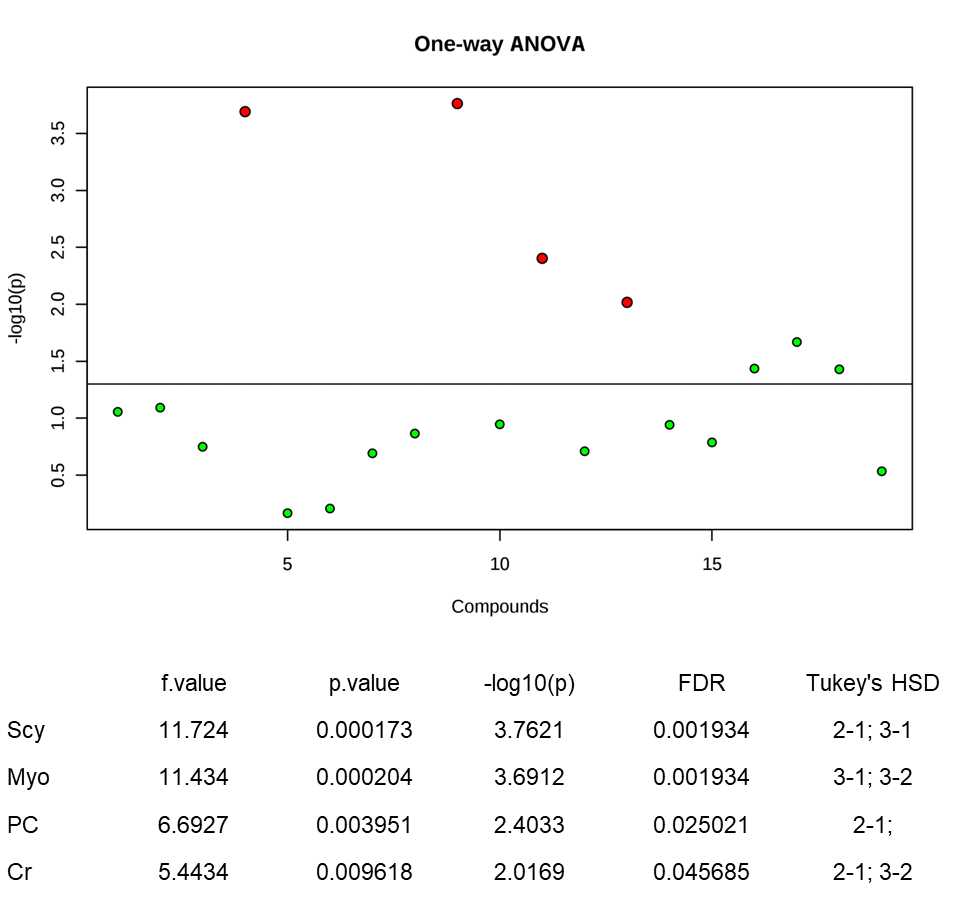


Figure S3. PLS-DA cross validation details.


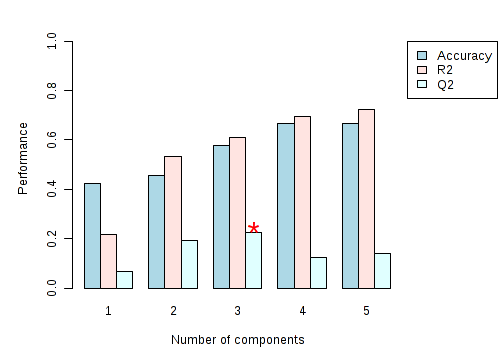


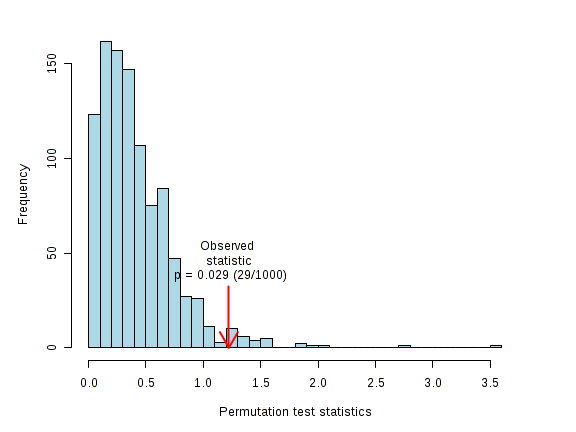

Supplement: Supplementary file 1 — Supporting Information [file 41598_2019_47984_MOESM1_ESM.docx]
